# Enzymatic Characterization of a Novel GH3 *β*-Glucosidase from *Lentilactobacillus buchneri*: EDTA-Mediated Enhancement of Thermostability

**DOI:** 10.3390/metabo16080565

**Published:** 2026-08-10

**Authors:** Hui Tang, Jinjian He, Can Li, Tongying Liu, Hao Wu, Mansheng Wang, Pengjun Shi

**Affiliations:** 1 Institute of Bast Fiber Crops, Chinese Academy of Agricultural Sciences, Changsha 410205, China; 18474334599@139.com (H.T.); hejinjian@caas.cn (J.H.); shipengjun@caas.cn (P.S.); 2 School of Food Science and Bioengineering, Changsha University of Science & Technology, Changsha 410114, China; lican@csust.edu.cn; 3 National Nanfan Research Institute, Chinese Academy of Agricultural Sciences, Sanya 572000, China; liutongying1019@163.com

**Keywords:** *Lentilactobacillus buchneri*, *β*-glucosidase, cold-adapted, EDTA, thermostability

## Abstract

Background/Objectives: β-Glucosidases from lactic acid bacteria are valuable biocatalysts for carbohydrate conversion, flavour enhancement, and bioactive glycoside biotransformation. Although GH3 family enzymes have been characterized from several Lactobacillus species, no systematic study exists for *Lentilactobacillus buchneri*—a GRAS strain with plant-polysaccharide-degrading potential. This work aimed to clone, heterologously express, and comprehensively characterize a novel GH3 β-glucosidase (*LbBgl3*) from *L. buchneri*, with a particular focus on its catalytic properties, substrate profile, stability, and the unexpected EDTA-mediated thermostabilization mechanism. Methods: A novel *β*-glucosidase gene (*LbBgl3*) from *L. buchneri* was successfully expressed in *Escherichia coli*. Results: Biochemical characterization revealed that *LbBgl3* is a cold-adapted, moderately acidophilic enzyme, exhibiting optimal activity at 37 °C and pH 5.0, with a maximum specific activity of 799.67 U·mg^−1^. Under optimal conditions, the enzyme displayed kinetic parameters toward *p*NPG with a *K*_m_ of 1.697 mM, *V*_max_ of 442.5 μmol·mg^−1^·min^−1^, *k*_cat_ of 634.32 s^−1^, and *k*_cat_/*K*_m_ of 373.79 mM^−1^·s^−1^. *LbBgl3* exhibited high salt tolerance, with activity peaking at ~140% at 0.5 M NaCl and retaining ~68% at 2.0 M NaCl, while showing sensitivity to glucose inhibition. Notably, EDTA significantly enhanced both the activity and stability of *LbBgl3*. At 50 mM, the relative activity increased to 161%, and stability at 25 °C was prolonged. Molecular docking simulations suggested that EDTA binds near the substrate-binding pocket, forming hydrogen bonds with Ala57, Gln766, Ser768, and Lys770, thereby stabilizing the local conformation and enhancing thermal resistance. Conclusions: This study presents the first characterization of a GH3 *β*-glucosidase from *L. buchneri* and reveals a non-classical stabilizing effect of EDTA, offering valuable insights for enzyme engineering and biocatalytic applications.

## 1. Introduction

Lactic acid bacteria (LAB) are generally recognized as safe (GRAS) probiotic starters cultures with diverse biological functions, including modulation of intestinal microbiota, enhancement of immune responses, and exertion of antioxidant and anti-inflammatory activities [1]. As dominant microorganisms in the intestinal tract, LAB inhibit pathogenic bacteria growth and promote the proliferation of probiotics such as *Bifidobacterium* spp., which together with other probiotics contribute to the formation of a protective barrier for intestinal health [2]. Notably, LAB possess unique metabolic capabilities and act as efficient biocatalysts in biotechnology, enabling the conversion of macromolecular carbohydrates and polysaccharides into value-added chemicals and functional foods [3]. The diverse metabolic capabilities of LAB are highly dependent on the versatility of their enzymatic systems, including glycoside hydrolases for carbohydrate metabolism [4], proteases for protein hydrolysis [5], as well as malic enzymes and lactate dehydrogenases involved in organic acid transformation [6].

Among these enzymes, glycoside hydrolases—particularly *β*-glucosidases (BGLs, EC 3.2.1.21) derived from *Lactobacillus* species—have garnered considerable attention due to their remarkable catalytic properties. These enzymes have been widely applied in multiple fields, such as the biotransformation of functional components [7], improvement of food flavor [8], and production of bioenergy [9]. For instance, the *β*-glucosidase Bgy1 from *L. brevis* catalyzes the conversion of ginsenosides Rb1, F2, and GypXVII into their low-molecular-weight derivatives (Rd, CK, and GypXXXV, respectively), which possess enhanced pharmaceutical value. In the food industry, LAB-derived *β*-glucosidases assume a crucial role in flavor modulation by hydrolyzing non-volatile, water-soluble glycosidic precursors naturally present in plant-based food matrices. This hydrolytic process releases a diverse array of aromatic compounds, including monoterpene alcohols (linalool, geraniol, nerol), C13-norisoprenoid derivatives, and aromatic alcohols (benzyl alcohol, phenethyl alcohol), collectively contributing to the distinctive aroma profiles of numerous food products. Furthermore, BGLs serve as rate-limiting enzymes in bioenergy production, catalyzing the key hydrolysis step of lignocellulosic biomass into fermentable sugars such as glucose and cellobiose—a depolymerization step essential for biofuel synthesis [10].

According to the Carbohydrate-Active Enzymes (CAZy) database, *β*-glucosidases are primarily categorized into two glycoside hydrolase (GH) families: GH1 and GH3. Most reported GH3 family *β*-glucosidases are of bacterial origin, including genera such as *Bacteroides*, *Bifidobacterium*, *Cellulomonas*, *Flavobacterium*, and *Lactobacillus* [11]. Research on probiotic-derived GH3 family *β*-glucosidases has focused primarily on *Bacteroides*, *Bifidobacterium*, and *Lactobacillus* [12]. Unlike the former two, GH3 family *β*-glucosidases from LAB typically exhibit optimal activity at 30–45 °C and pH 5.0–7.5, indicating psychrotolerant and acidophilic characteristics. Furthermore, structural domains variations confer distinct functionalities, enabling applications in the biotransformation of glycosidic bioactive components such as ginsenosides, soy isoflavones, prunasin, and geniposide. For example, LpBgla from *L. paracasei* TK1501, with optimal activity at 30 °C and pH 5.5, specifically hydrolyzes geniposide, salicin, polydatin, esculin, and arbutin to generate low-molecular-weight bioactive components [13]. Similarly, BaBgl3A from *B. adolescentis* ATCC 15703 shows optimal activity at 45 °C and pH 6.5, exhibits specific hydrolytic activity toward *β*-1,3 and *β*-1,4-glycosidic bonds, and has been used to convert ginsenoside Rb1 to Rd and GypXVII [14]. Additionally, a *β*-glucosidase from *L. casei* SC1, with optimal conditions of 30 °C and pH 7.5, effectively hydrolyzes daidzin [15]. These findings collectively establish LAB as a rich source of *β*-glucosidase biocatalysts, with considerable promise for future exploration of high-performance enzymes.

Despite the promising applications of *Lactobacillus*-derived *β*-glucosidases, structural and functional investigation of enzymes from the genus *Lentilactobacillus* remains limited. As the type strain of this genus, *L. buchneri* has been approved by the European Union as a silage additive due to its ability to reduce fiber content in silage [16]. Such carbohydrate degradation may be mediated by β-glucosidases produced by *Lentilactobacillus* species. However, the lack of comprehensive structural and functional data on *L. buchneri-*derived *β*-glucosidases has considerably hindered in-depth exploration of the molecular mechanisms underlying their biotransformation of bioactive components. In this study, a novel GH3 family *β*-glucosidase, designated *LbBgl3*, from *L. buchneri* was heterologously expressed in *Escherichia coli*, and its enzymatic characteristics and amino acid sequence sites affecting thermostability were investigated.

## 2. Materials and Methods

### 2.1. Bacterial Strains and Chemicals

*E. coli* BL21(DE3) (General, Hefei, Anhui, China) was used as the host for expressing the *LbBgl3* gene. Gentiobiose, Sophorose and 4-Nitrophenyl-*β*-D-xylopyranoside (*p*NPX) were purchased from Megazyme (Bray, Ireland). *p*-Nitrophenyl-*β*-D-glucopyranoside (pNPG), *p*-Nitrophenyl-*β*-D-galactopyranoside (*p*NPGal), and *o*-Nitrophenyl-*β*-D-galactopyranoside (*o*NPGal) were also obtained from Megazyme. All reagents were analytically pure unless otherwise specified.

### 2.2. Bioinformatics Analysis

The target protein sequence was analyzed using the BLAST program on the NCBI database (https://blast.ncbi.nlm.nih.gov). Multiple sequence alignment was performed using Clustal W (https://www.genome.jp/tools-bin/clustalw, accessed on 2 September 2025). Amino acid composition analysis was performed via the Expasy ProtParam (https://web.expasy.org/protparam/). The domain architecture of *LbBgl3* was analyzed with the SMART program (http://smart.embl-heidelberg.de, accessed on 22 December 2025). Structural visualization was conducted with PyMOL 2.6.0a0 (Open-Source Build) software.

### 2.3. Expression and Purification of LbBgl3

The recombinant strain *E. coli* BL21(DE3)-pET-30a(+)-*LbBgl3* was activated by overnight shaking at 37 °C and 220 rpm. The culture was then incubated until the OD_600_ reached 0.4–0.8. Isopropyl-*β*-D-1-thiogalactopyranoside (IPTG) was added to the kanamycin-supplemented LB medium to a final concentration of 0.6 mM, and protein expression was induced by incubation overnight at 16 °C and 220 rpm. After induction, bacterial cells were harvested by centrifugation at 9028× *g* for 10 min. The collected cells were resuspended in lysis buffer (20 mM Tris-HCl, 500 mM NaCl, pH 7.0) and disrupted by ultrasonication (Power: 300 W, working time: 6 s, interval time: 10 s, total crushing time: 25 min). The cell lysate was centrifuged at 9028× *g* for 10 min at 4 °C to remove cell debris, and the supernatant was collected. The purified recombinant *LbBgl3* solution was stored at 4 °C until further use. Recombinant *LbBgl3* was purified from the supernatant using Ni-immobilized metal affinity chromatography (Ni-IDA) magnetic beads. Protein purity and molecular weight were assessed by 12% sodium dodecyl sulfate-polyacrylamide gel electrophoresis (SDS-PAGE), and protein concentration was quantified using a bicinchoninic acid (BCA) protein assay kit.

### 2.4. Enzyme Assay

The activity of recombinant *β*-glucosidase *LbBgl3* was determined using *p*NPG as the substrate. The reaction system consisted of 250 μL of 4 mM *p*NPG and 250 μL of appropriate concentration purified enzyme solution, incubated at 35 °C for 10 min. The final concentration of purified *LbBgl3* in the reaction system was 0.5 μg/mL, as determined by BCA protein assay. The reaction was terminated by adding 1.5 mL of 1 M Na_2_CO_3_. The absorbance of the released *p*-nitrophenol (*p*NP) was measured at 405 nm using a UV-visible spectrophotometer. Three biological replicates were performed for each experiment, and a control (with termination solution added before the enzyme) was included to account for non-enzymatic hydrolysis.

The hydrolytic activities of *LbBgl3* towards disaccharide substrates (cellobiose, sophorose and gentiobiose) were determined using the glucose oxidase-peroxidase (GOD-POD) method. The absorbance was measured at 520 nm, and the amount of released glucose was quantified using a glucose standard curve.

One unit (U) of *β*-glucosidase activity was defined as the amount of enzyme required to hydrolyze 1 μmol of *p*NPG (or glycoside substrates) and release 1 μmol of *p*NP (or glucose) per minute under the aforementioned reaction conditions.

### 2.5. Optimal Catalytic Conditions and Thermal Stability

The optimal temperature for *LbBgl3* activity was determined by assaying activity over a range of 0–50 °C under the standard reaction conditions. The optimal pH was determined using citrate-phosphate buffer (pH 4.5–8.0) at the optimal temperature. The highest relative activity was defined as 100%.

The thermostability was assessed by incubating purified enzyme solutions at 25, 30, and 37 °C for 0, 10, 20, 30, and 60 min, respectively. At each time point, samples were collected, and their residual enzyme activities were assayed under the pre-determined optimal reaction conditions. The non-incubated original enzyme solution was used as a control (with its activity set as 100%), and the relative enzyme activity at each time point was calculated to evaluate the thermostability of *LbBgl3*.

### 2.6. Assay of Substrate Specificity and Kinetic Constants

In order to comprehensively elucidate the substrate specificity of *LbBgl3*, the enzymatic activity was systematically evaluated under the pre-established optimal catalytic conditions. A series of substrates, including artificial substrates *p*NPG, *p*NPX, *p*NPGal and natural disaccharide substrates (cellobiose, gentiobiose, sophorose), were employed at a concentration of 4 mM, respectively. For artificial substrate hydrolytic activity assessment, the *p*NPG-based quantification assay was performed following established protocols. Regarding the natural disaccharide substrates, the GOD-POD method was utilized to determine the hydrolytic activity.

The kinetic parameters of *LbBgl3* with respect to *p*NPG were meticulously determined under the optimal reaction conditions. Specifically, the enzymatic activity assays were conducted using *p*NPG at final concentrations of 0.25, 0.5, 1.0, 2.0, and 4.0 mM. Following a 10-min reaction period, the absorbance at 405 nm was precisely recorded. The Michaelis constant (*K*_m_), maximum reaction velocity (*V*_max_), turnover number (*k*_cat_), and catalytic efficiency (*k*_cat_/*K*_m_) were calculated by nonlinear regression fitting of the Michaelis–Menten equation. Given the high homogeneity of purified *LbBgl3* verified by native electrophoresis, the active fraction was regarded as 100%.

$$k_{cat}=\frac{V_{max}\cdot M}{60}$$

where *V*_max_ is the maximum reaction rate (μmol·mg^−1^·min^−1^), M is the molecular mass of *LbBgl3* (kDa), and division by 60 converts units from min^−1^ to s^−1^. All assays were performed in triplicate. All assays were performed in triplicate.

### 2.7. The Influence of Metal Ions and Chemical Reagents on LbBgl3

Using *p*NPG as the substrate, the effects of metal ions (K^+^, Ca^2+^, Zn^2+^, Mg^2+^, Mn^2+^, Fe^2+^, Fe^3+^, Cu^2+^, Na^+^, Pb^2+^, Ni^2+^, Li^+^, Cd^2+^, Co^2+^), the inhibitor SDS, the chelator EDTA, and organic solvents (methanol, isopropanol, *β*-mercaptoethanol) on *LbBgl3* activity were investigated. Metal ions, SDS, and EDTA were added to a final concentration of 5 mM. Organic solvents were added to a final concentration of 5% (*v*/*v*). Relative activity was determined as described above, with each condition tested in triplicate.

### 2.8. Influences of Additives on Enzyme Activity

The tolerance of *LbBgl3* to methanol, ethanol, glucose, NaCl, and EDTA was evaluated by adding these compounds to the standard reaction system. The control group without additives was set to 100% relative activity. Methanol and ethanol were tested at final concentrations of 5%, 10%, 15%, 20%, and 25% (*v*/*v*). Glucose and NaCl were tested at final concentrations of 0.5, 1.0, 1.5, and 2.0 M. EDTA was tested at final concentrations of 5, 25, 50, 100, 200, and 250 mM. After incubation of each reaction system under the established optimal catalytic conditions, the relative enzymatic activity of *LbBgl3* was determined using the *p*NPG-based assay method described previously, with each concentration tested in triplicate.

### 2.9. Three-Dimensional Modeling and Molecular Docking

The three-dimensional structure of *LbBgl3* was predicted using AlphaFold3, and the quality of the predicted structure was evaluated using Venn diagrams. Molecular docking between *LbBgl3* and the ligands (EDTA) was performed with AutoDockTools 1.5.7 (Scripps, La Jolla, CA, USA), where the docking box was set to 50 Å × 50 Å × 50 Å with a grid spacing of 0.375 Å. The center coordinates of the substrate binding pocket (−9.7, 19.14, 9.01) were predicted using Deepsite (https://open.playmolecule.org/tools, accessed on 21 January 2026) [17] and used as the center coordinates for the molecular docking box. This study used the H_2_Y^2−^ form of EDTA (derived from Na_2_EDTA) for docking at pH 5.0. For molecular docking simulations, the optimal binding conformation was screened from all generated poses according to minimal binding energy, absence of steric clashes, and occupancy within the substrate-binding pocket. Hydrogen bonds were defined using a heavy-atom distance cutoff ≤ 3.6 Å and a marginal hydrogen bond angle threshold of 63.0°.

### 2.10. Isothermal Titration Calorimetry (ITC) Measurement

ITC experiments were performed at 25 °C (298 K) using a NANO ITC calorimeter (TA Instruments, New Castle, DE, USA), following the experimental design described in previous literature [18]. The experimental parameters were set as follows: Macromolecule Binding Active (MBA, the concentration of active macromolecules capable of binding) was 0.05 mM, Macromolecule in Cell (MB, the concentration of macromolecules in the reaction cell) was 0.175 mM, and Constant Dilution Parameter (CDP, the constant dilution heat parameter) was 0.01 mM; all reagents were pretreated before the experiments. For sample loading and titration parameters, 0.05 mM MBA and 0.175 mM MB were separately aspirated into the titration syringe with an initial volume of 50 μL for each, and the CDP-related system was added to the sample cell with an initial volume of 300 μL. The titration procedure consisted of 20 consecutive injections, with a single injection volume of 2 μL and an interval of 120 s between adjacent injections; the stirring speed of the syringe was set at 350 r/min during the titration. Two groups were set up in the experiment: the experimental group (MBA/MB titrated against the CDP-related system) and the control group (MBA/MB titrated against blank solvent). The operation procedures and experimental parameters were identical for both groups, and the data from the blank titration were used to obtain the CDP value. All experimental and control groups were conducted in duplicate to ensure the reliability and reproducibility of the results.

After the experiments, the obtained isotherms were integrated and the blank heat effects from the control group were subtracted. Global fitting was performed using the 1:1 heteroassociation model (A + B→AB), and the binding stoichiometry (*n*), dissociation constant (*K_d_*), entropy change (Δ*S*), and enthalpy change (Δ*H*) were calculated by incorporating the MBA, MB, and CDP parameters. The fitted data were processed using Origin 2021 software to generate the experimental plots meeting the requirements of academic journal publication.

### 2.11. Statistical Analysis

All experiments were performed at least three times, and the data are presented as mean ± standard deviation (SD). Statistical analysis and graphing were performed using Microsoft Excel 2019 (Microsoft Corporation, Redmond, WA, USA) and Adobe Illustrator 2022 (Adobe Systems Incorporated, San Jose, CA, USA).

## 3. Results

### 3.1. Sequence and Structural Analysis

**Figure 1:**
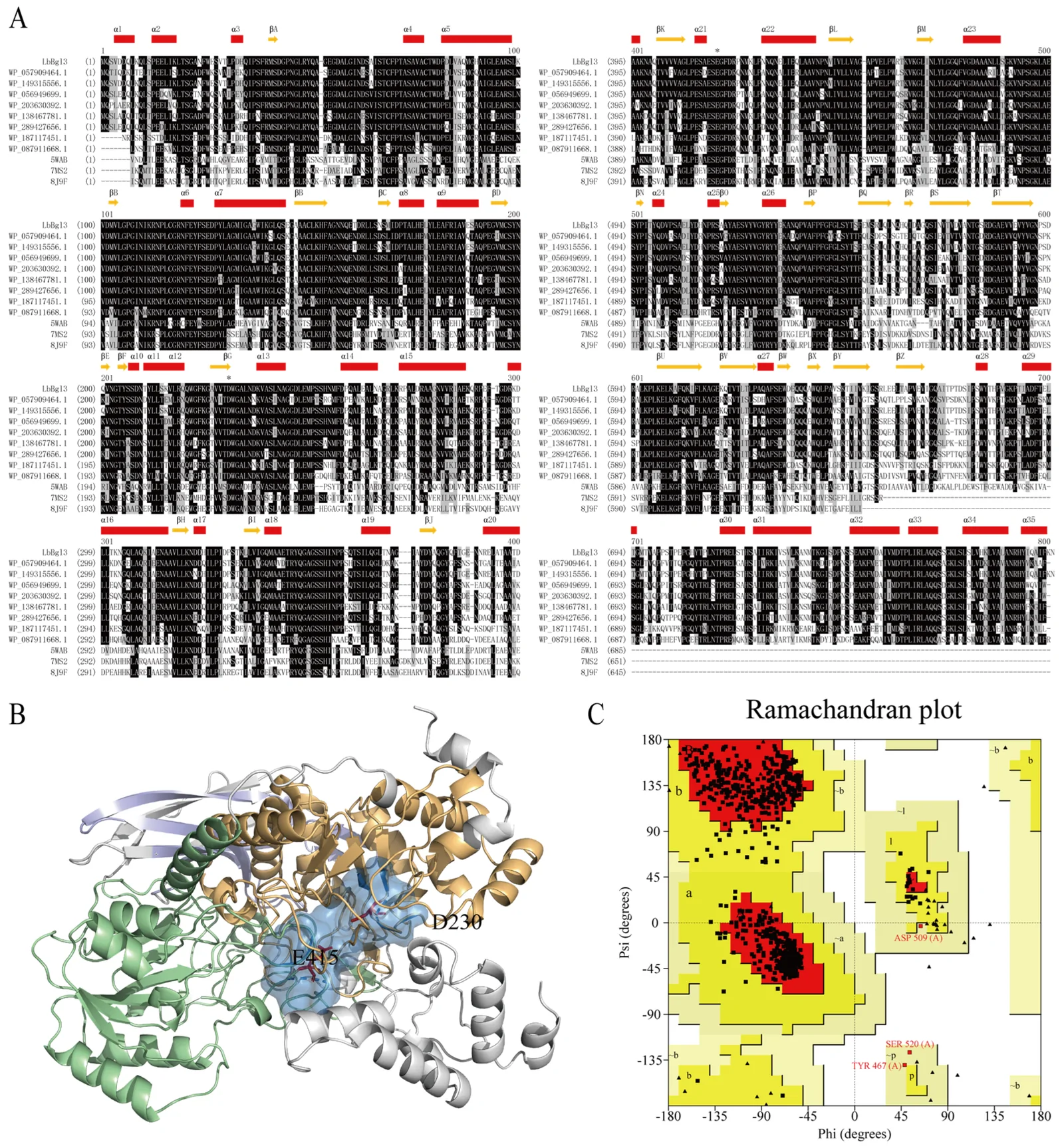
Sequence and structural analysis of *LbBgl3*. (**A**) Multiple sequence alignment of *LbBgl3* with homologs. The homologue set consisted of four *β*-glucosidases from *Lentilactobacillus*, including *LbBgl3* and three homologous enzymes (NCBI accession numbers: WP_149315556.1, WP_056949699.1, and WP_203630392.1), along with three *β*-glucosidases from the GH3 family with resolved crystal structures (PDB IDs: 5WAB, 7MS2, and 8J9F). (**B**) Domain architecture and catalytic residues of *LbBgl3*. The (α/*β*)_8_ barrel, (α/*β*)_6_ sandwich, and FnIII domains are shown in light orange, pale green, and light blue, respectively. Catalytic residues D230 and E415 are highlighted as red sticks. (**C**) Ramachandran map of *LbBgl3*.

The gene encoding a GH3 family *β*-glucosidase from *L. buchneri* was retrieved from the NCBI database (GenBank No: CAM3048833.1). Sequence analysis revealed a 2385 bp open reading frame encoding a 794-amino acid polypeptide, designated *LbBgl3*. BLAST analysis indicated that *LbBgl3* shares the highest sequence identity (99.87%) with a *β*-glucosidase from *L. buri* (NCBI Reference Sequence: WP_149315556.1), followed by 78.41% identity with an enzyme from *L. kisonensis* (GenBank: AZEB01000046.1) and 43.80% identity with a structurally characterized GH3 *β*-glucosidase (Protein Data Bank, PDB: 7MS2). Multiple sequence alignment identified highly conserved catalytic residues: aspartic acid (D230) in the T**D**WG motif and glutamic acid (E415) in the ES**E**GF motif (Figure 1A). Importantly, the distribution pattern of these conserved catalytic residues and their corresponding motif locations exhibits a remarkable degree of congruence with those of previously reported GH3 family *β*-glucosidases [19].

The three-dimensional structure of *LbBgl3*, predicted by AlphaFold3, was of high quality (Figure 1B). Structural analysis revealed that *LbBgl3* comprise the typical GH3 family structural domains, including a (*β*/α)_8_ barrel domain (residues 18–284), an (α/*β*)_6_ sandwich domain (residues 315–545), and a fibronectin type III (FnIII) domain (residues 582–652). The catalytic residue D230 is localized within the (*β*/α)_8_ barrel domain, while E415 is positioned at the interface between the (*β*/α)_8_ barrel and (α/*β*)_6_ sandwich domains, with a distance of approximately 6.0 Å between them (Figure 1B). FnIII domains are typical domains of GH3 family enzymes, but their specific functions remain unclear. Additionally, DeepSite prediction identified three potential substrate-binding pockets, among which the pocket within the (*β*/α)_8_ barrel domain was most consistent with the expected catalytic conformation (Figure 1B).

The Ramachandran plot (Figure 1C) remains as a complementary validation metric, showing that 96.4% of residues are in favored regions, further confirming the structural reliability. In addition, the AlphaFold3-predicted structure achieved a mean pLDDT score of 88.6, indicating high overall confidence, with the catalytic core domains exhibiting the highest scores (>90).

### 3.2. Expression and Purification of Recombinant LbBgl3

**Figure 2:**
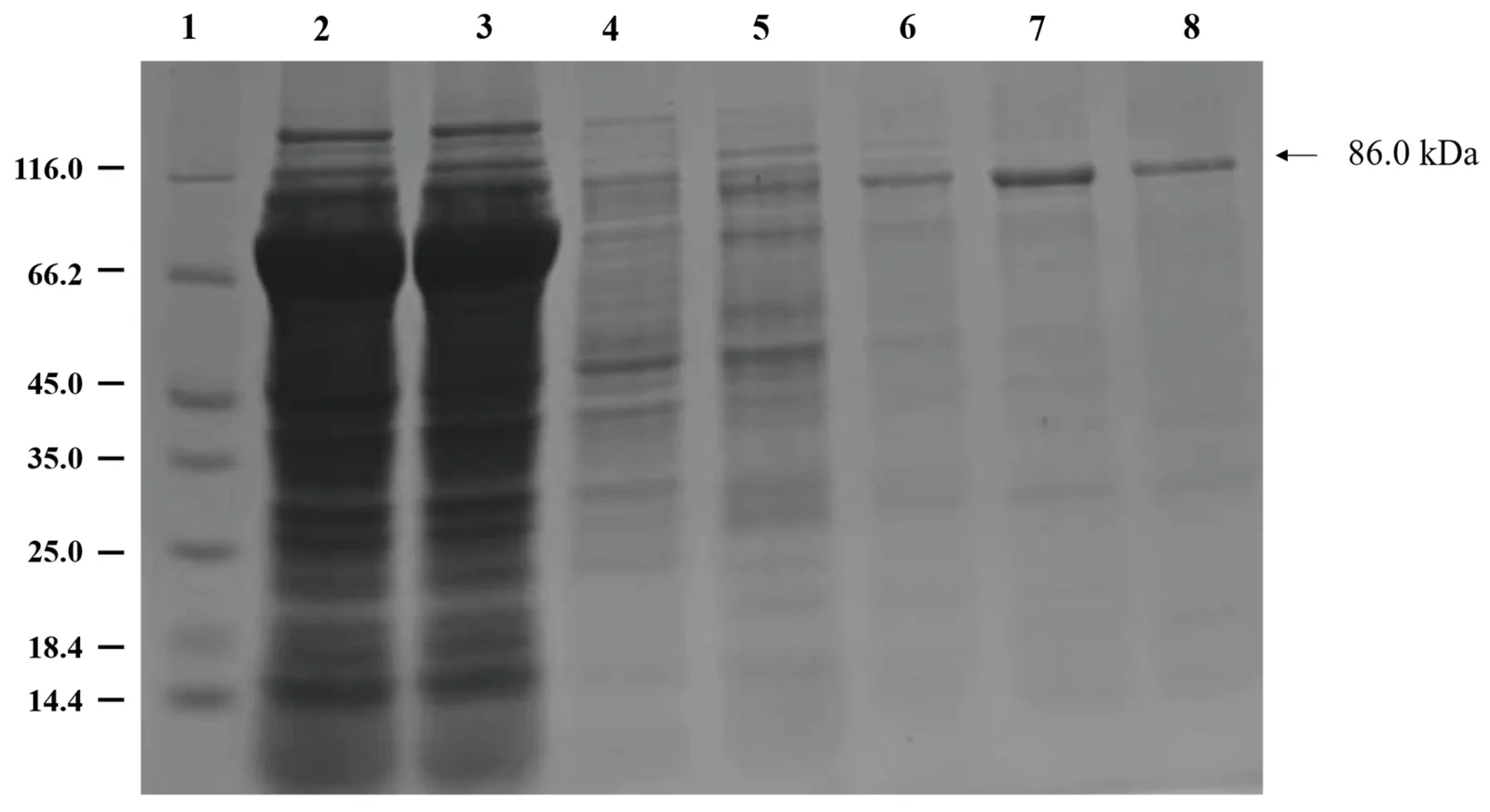
SDS-PAGE of *LbBgl3* expression and purification. Lane 1: Marker; Lane 2: Cell lysates supernatant. Lane 3: Waste liquid without combined magnetic beads; Lane 4: 0 mM imidazole elution buffer. Lane 5: 40 mM imidazole elution buffer. Lane 6: 100 mM imidazole elution buffer. Lane 7: 200 mM imidazole elution buffer. Lane 8: 300 mM imidazole elution buffer.

Recombinant *LbBgl3*, containing an N-terminal 6 × His tag, was successfully expressed in *E. coli* BL21(DE3) and purified using Ni-IDA magnetic beads. The theoretical molecular weight was calculated to be 86.0 kDa. SDS-PAGE analysis of the purified protein showed a single band with an apparent molecular weight of approximately 86.0 kDa (Figure 2), confirming successful expression and purification to homogeneity.

### 3.3. Enzymatic Properties of Recombinant LbBgl3

#### 3.3.1. Optimal Temperature and pH

**Figure 3:**
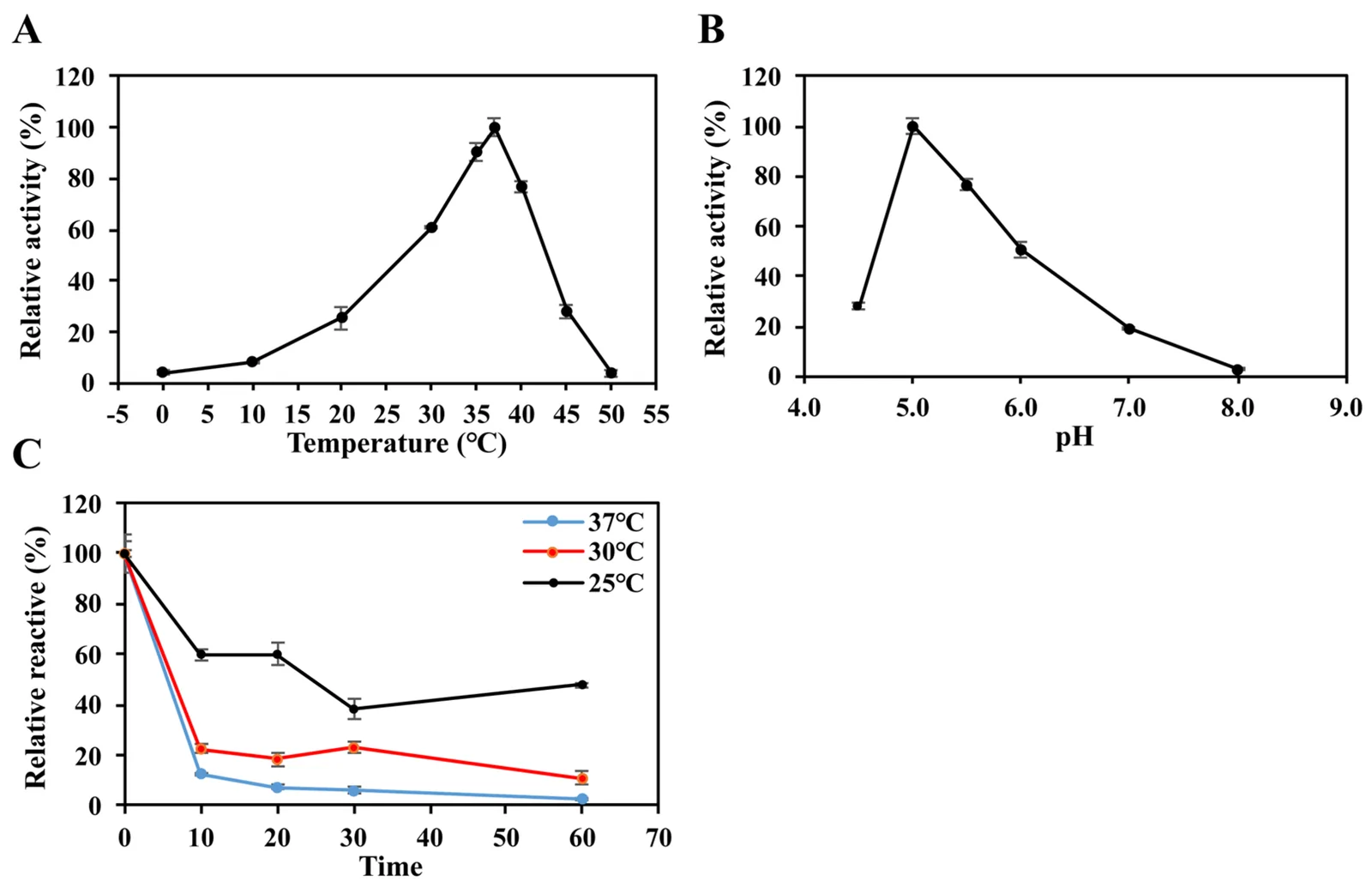
Optimal catalytic conditions and thermostability of *LbBgl3*. (**A**) Optimal temperature. (**B**) Optimal pH. (**C**) Thermostability of *LbBgl3* at 25 °C, 30 °C, and 37 °C in pH 5.0 buffer. Data are presented as mean ± SD from three independent biological replicates (n = 3).

Recombinant *LbBgl3* exhibited maximum activity at 37 °C and pH 5.0 (Figure 3A,B). This enzyme is an acidophilic cold-adapted [20] enzyme and shares enzymatic properties similar to those of typical *β*-glucosidases derived from *Lactobacillus* species (Table 1). *LbBgl3* displayed pronounced temperature sensitivity, characterized by relatively high retention of catalytic activity at low temperatures and rapid inactivation at moderate to high temperatures. Specifically, it retained over 60% of its initial activity within the temperature range of 30–40 °C, while maintaining detectable activity at lower temperatures (≤25 °C). In contrast, a sharp decline in catalytic activity was observed when the temperature exceeded 45 °C, with nearly complete inactivation at 50 °C. This temperature-dependent pattern is consistent with the characteristics of cold-adapted enzymes. Furthermore, thermostability characterization of *LbBgl3* demonstrated poor thermoresilience. Upon incubation at 37 °C for 10 min, the enzyme exhibited less than 13% of its initial activity (Figure 3C). Conversely, when incubated at 25 °C for the same duration, approximately 60% of its initial relative activity was retained (Figure 5B), further confirming its typical cold-adapted enzyme features [20]. Comparative analysis with *β*-glucosidases classified as cold-adapted, mesophilic, and thermophilic [21] revealed significant differences in the amino acid composition of *LbBgl3* (Table 2). Specifically, marked compositional characteristics unique to cold-adapted enzymes, particularly *LbBgl3*, were identified by comparing amino acid profiles of cold-adapted *LbBgl3* and M-GH3_A from *Marinomonas* sp. ef1 [22], mesophilic MtBgl85 derived from *Microbulbifer thermotolerans* DAU221 [23], and thermophilic Bgl3B originating from *Talaromyces leycettanus* [24]. As a typical cold-adapted β-glucosidase, *LbBgl3* accumulates high levels of Leu, Lys and Gln but contains less rigid Arg and aromatic Trp relative to mesophilic and thermophilic counterparts; this composition weakens intramolecular salt bridges and hydrophobic stacking, endowing the active pocket with adequate conformational flexibility to sustain catalytic efficiency under low-temperature conditions. Compared with another cold-adapted ortholog M-GH3_A, *LbBgl3* features elevated Ala and Pro contents, which moderately strengthens backbone rigidity and avoids excessive structural looseness induced by low temperature. Distinct from mesophilic MtBgl85 rich in stabilizing Ala and Arg and thermostable Bgl3B enriched in Gly, Val, Trp and Asp for robust thermal resistance, *LbBgl3* adopts a cold-adaptive strategy that minimizes rigid intramolecular interactions to prioritize structural flexibility, a representative molecular signature supporting its high catalytic activity in cold environments.

*LbBgl3* shows a preference for acidic environments as its optimal pH. Its catalytic activity can be detected across a wide pH span from 3.0 to 7.0, demonstrating that it is a moderately acidophilic *β*-glucosidase. The optimal activity of this enzyme precisely mirrors the physiological growth conditions of its native host, *L. buchneri*. Notably, *L. buchneri* thrives optimally within the temperature range of 15–37 °C and remains viable even at a low pH of 4.4 [30,31]. From an evolutionary perspective, the adaptation of *LbBgl3* in *L. buchneri* represents a sophisticated response to its cold and acidic ecological niche. The congruence between the enzyme’s optimal catalytic conditions at 37 °C and pH 5.0 and its native habitat further validates its functional suitability.

#### 3.3.2. Substrate Specificity and Kinetic Constants

**Figure 4:**
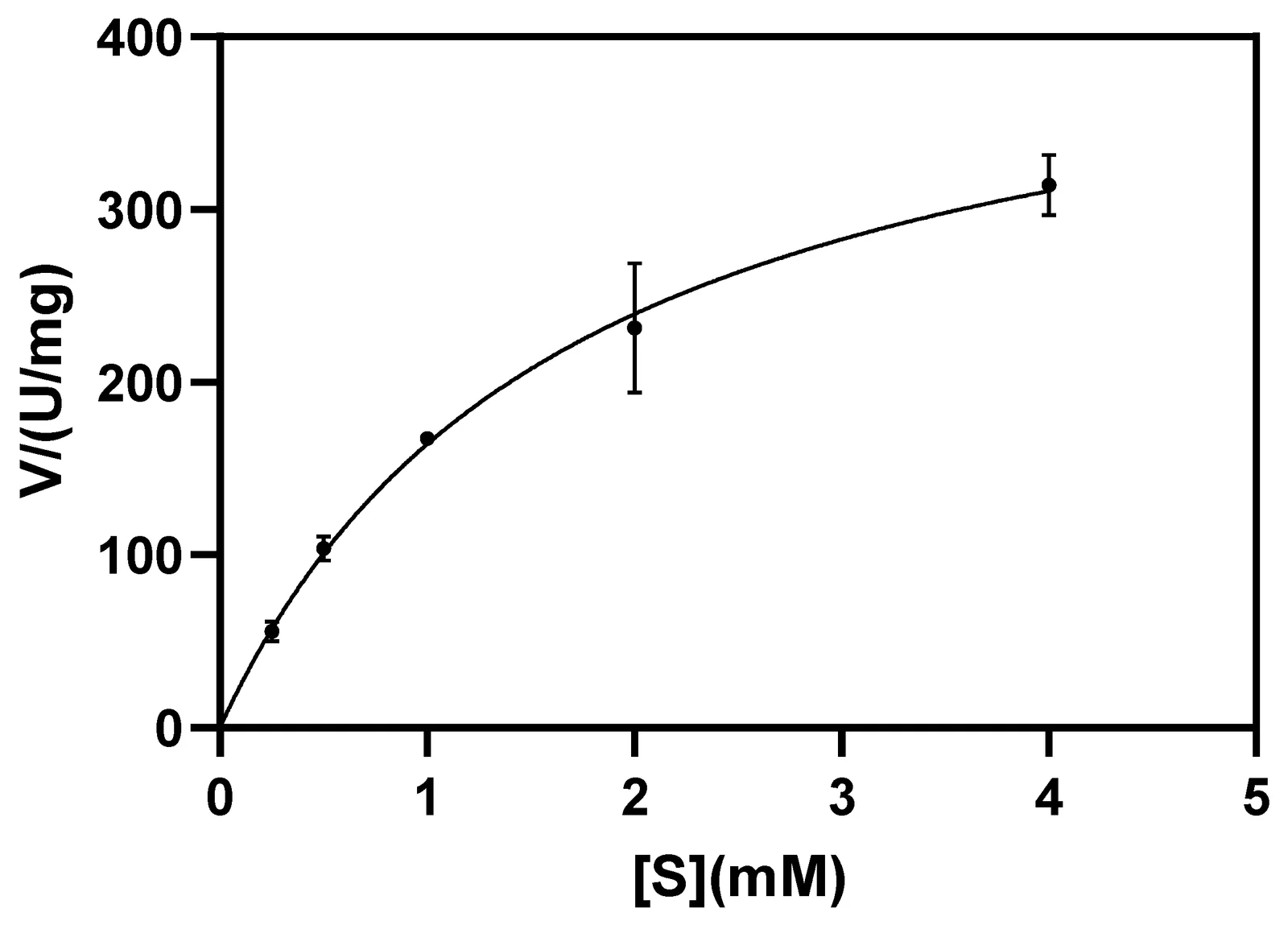
Nonlinear Michaelis-Menten fitting curve. [S] represents the substrate concentration. Data are presented as mean ± SD from three independent biological replicates (n = 3).

Under optimal conditions (37 °C, pH 5.0), the kinetic parameters of *LbBgl3* toward *p*NPG were determined (Figure 4). Nonlinear regression fitting was performed in conjunction with the Michaelis–Menten equation to determine enzymatic kinetic parameters., with *K*_m_, *V*_max_, *k*_cat_ and *k*_cat_/*K*_m_ calculated to be 1.697 mM, 442.5 μmol·mg^−1^·min^−1^, 634.32 s^−1^, and 373.79 mM^−1^·s^−1^, respectively. Substrate specificity assays revealed that *LbBgl3* exhibited exceptionally high specific activity towards *p*NPG, but showed no detectable hydrolysis of the disaccharide substrates (cellobiose, sophorose, gentiobiose) or other aryl *β*-glycosides under the tested conditions. Reported GH3 family *β*-glucosidases from *Lactobacillus* species generally show low specific activities toward *p*NPG, ranging from 0.25 to 150 U·mg^−1^ [7,13,25,26]. Notably, *LbBgl3* demonstrates a significantly elevated specific activity toward *p*NPG, reaching a peak value of 799.67 U·mg^−1^. This activity level is substantially higher than that of other homologous *β*-glucosidases isolated from *Lactobacillus* species (Table 1). A similar phenomenon has been reported for HiBgl3B, a GH3 *β*-glucosidase from *Humicola insolens* Y1, which also exhibits activity exclusively toward aryl *β*-glucosides (*p*NPG) and fails to hydrolyze cellobiose, sophorose, and gentiobiose. Xia et al. demonstrated that this narrow specificity is determined by three residues (Ile48, Ile278, Thr484) at the +1 subsite that form a constricted binding pocket incapable of accommodating the second glucosyl moiety of disaccharides [32]. Site-directed mutagenesis replacing these residues with the Trp/Phe/Tyr triad conserved in broad-spectrum GH3 enzymes (HiBgl3A) conferred hydrolytic activity toward sophorose, confirming the critical role of the +1 subsite architecture in substrate selectivity. By analogy, the narrow substrate profile of *LbBgl3* may similarly arise from restricted +1 subsite features, which could be tested by comparative structural analysis or mutagenesis in future studies.

The inability of *LbBgl3* to hydrolyze common *β*-linked disaccharides such as cellobiose, gentiobiose, and sophorose suggests that its natural substrate may be a plant-derived β-glucoside or glucoconjugate present in silage materials, consistent with the ecological niche of its native host *L. buchneri*. This substrate specificity pattern warrants further investigation through substrate screening with a more comprehensive glycoside library.

#### 3.3.3. Effect of Solvent, Salt and Glucose on Enzymatic Activity

**Figure 5:**
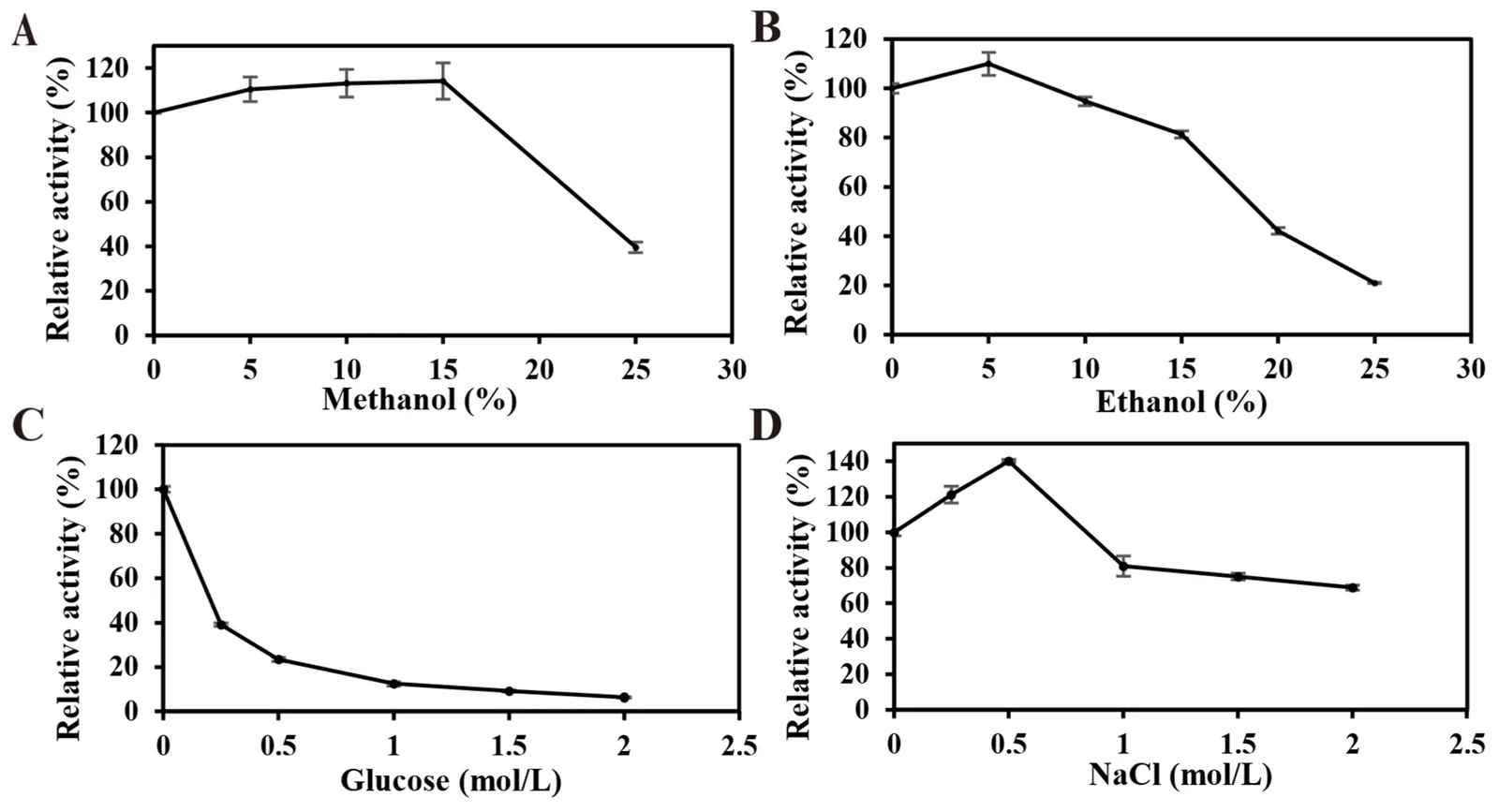
The solvent tolerance of *LbBgl3*. (**A**) Methanol tolerance. (**B**) Ethanol tolerance. (**C**) Glucose tolerance. (**D**) NaCl tolerance. Data are presented as mean ± SD from three independent biological replicates (n = 3).

The effects of methanol, ethanol, NaCl, and glucose on *LbBgl3* activity are shown in Figure 5. Enzymatic activity initially increased and then decreased with increasing concentrations of methanol or ethanol. At low concentrations, both alcohols slightly enhanced activity. Notably, in the presence of 15% (*v*/*v*) methanol, the enzyme still retained 114% of its relative activity, suggesting its potential applicability in the transformation of alcohol-soluble glycosides such as ginsenosides. Salt tolerance followed a similar trend, with maximum activity (~140%) observed at 0.25–0.50 M NaCl. In contrast, *LbBgl3* exhibited poor glucose tolerance, a characteristic commonly observed among GH3 family *β*-glucosidases. Overall, *LbBgl3* represents a novel *β*-glucosidase with tolerance to low concentrations of alcohols and NaCl.

#### 3.3.4. The Influence of Metal Ions and Chemical Reagents on the Activity of *LbBgl3*

The effects of various metal ions and chemical reagents on *LbBgl3* activity are summarized in Table 3. Specifically, Mg^2+^ and Zn^2+^ had no significant effect. In contrast, K^+^, Cu^2+^, Mn^2+^, Pb^2+^, Fe^2+^, Fe^3+^, sodium dodecyl sulfate (SDS), isopropanol, and *β*-mercaptoethanol exhibited inhibitory effects on *LbBgl3* activity. Among these inhibitors, Fe^2+^ and Fe^3+^ caused severe inhibition, reducing the relative activity of *LbBgl3* to less than 15%. Additionally, Na^+^, Li^+^, Cd^2+^, Co^2+^, Ni^2+^, EDTA, methanol and ethanol displayed stimulatory effects on *LbBgl3*, leading to an increased catalytic activity compared to the control.

**Figure 6:**
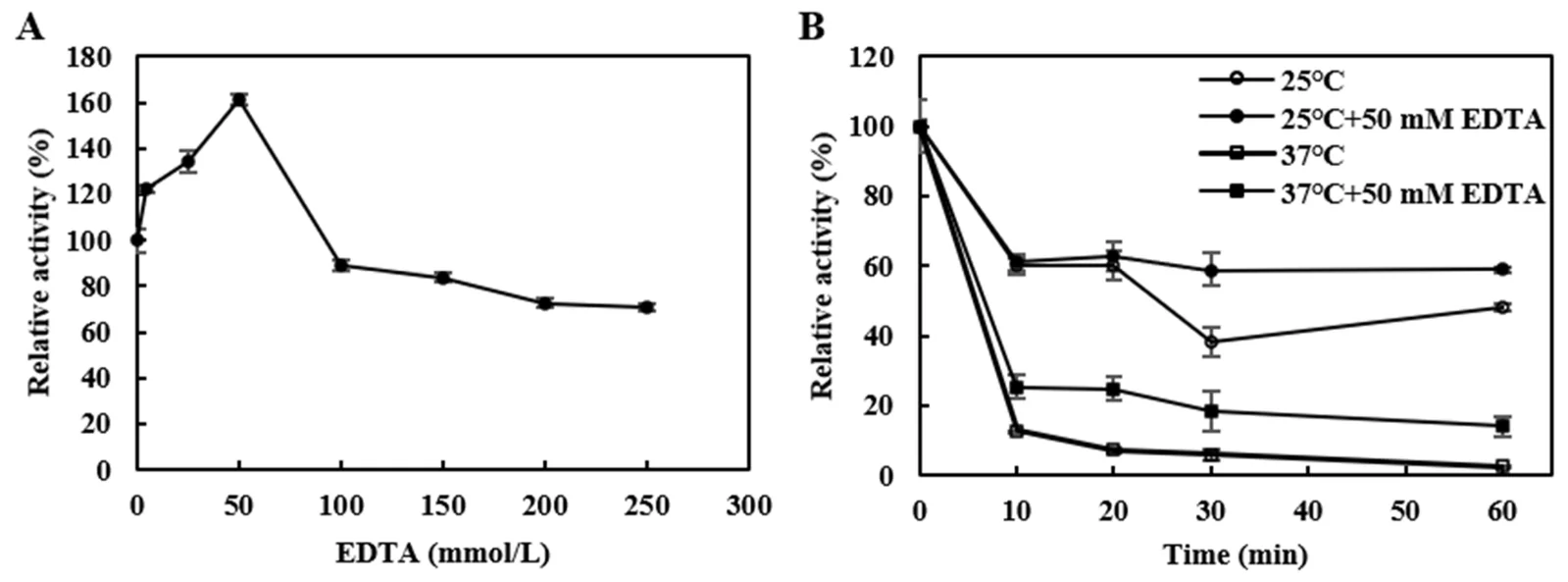
The effect of EDTA on the enzymatic activity of *LbBgl3*. (**A**) Effect of EDTA concentration on *LbBgl3* activity measured under standard assay conditions. (**B**) Thermostability of *LbBgl3* at 25 °C and 37 °C in pH 5.0 buffer with or without 50 mM EDTA. Residual activities were determined under optimal conditions. Data are presented as mean ± SD from three independent biological replicates (n = 3). Detailed experimental procedures are described in Section 2.5 and Section 2.8.

Notably, the metal chelator EDTA enhanced *LbBgl3* activity to 122% of the control at 5 mM, which contrasts with most reported *β*-glucosidases that typically is inhibited by EDTA. A gradual increase in *LbBgl3* activity was observed as the EDTA concentration was raised from 5 mM to 50 mM, reaching 161%. Conversely, a sharp decline in enzymatic activity occurred when the EDTA concentration was increased to 100 mM; nevertheless, 71% relative activity was retained even at 250 mM (Figure 6A). Although no studies have to date reported EDTA-induced enhancement of *β*-glucosidase activity, a similar phenomenon has been documented in other non-metal-dependent enzymes. For instance, in the presence of 5 mM EDTA, the melting temperature (T_m_) of endoglucanase from *Aspergillus aculeatus* increased by 16 °C, and mechanistic studies confirmed that EDTA improved enzyme activity by increasing *k*_cat_ and enhancing the conformational flexibility [33]. Additionally, a similar phenomenon was observed in cyclomaltodextrinase from *Geobacillus thermopakistaniensis*, where EDTA enhanced enzyme thermostability, thereby improving catalytic activity [34]. These findings suggest that low-concentrations of EDTA may stabilize the conformation of *LbBgl3*, which effectively prolongs the thermal inactivation half-life of the enzyme and simultaneously elevates its catalytic activity. This hypothesis is supported by thermostability assays of *LbBgl3* in the presence of EDTA: after heat treatment at 25 °C for 60 min, the activity of EDTA-supplemented *LbBgl3* remained comparable to that observed after heat treatment for 10 min (Figure 6B,C). This minimal activity loss over extended heating directly reflects the extended thermal inactivation half-life conferred by EDTA supplementation.

### 3.4. In Silico Analysis of EDTA-Mediated Thermostability Enhancement

**Figure 7:**
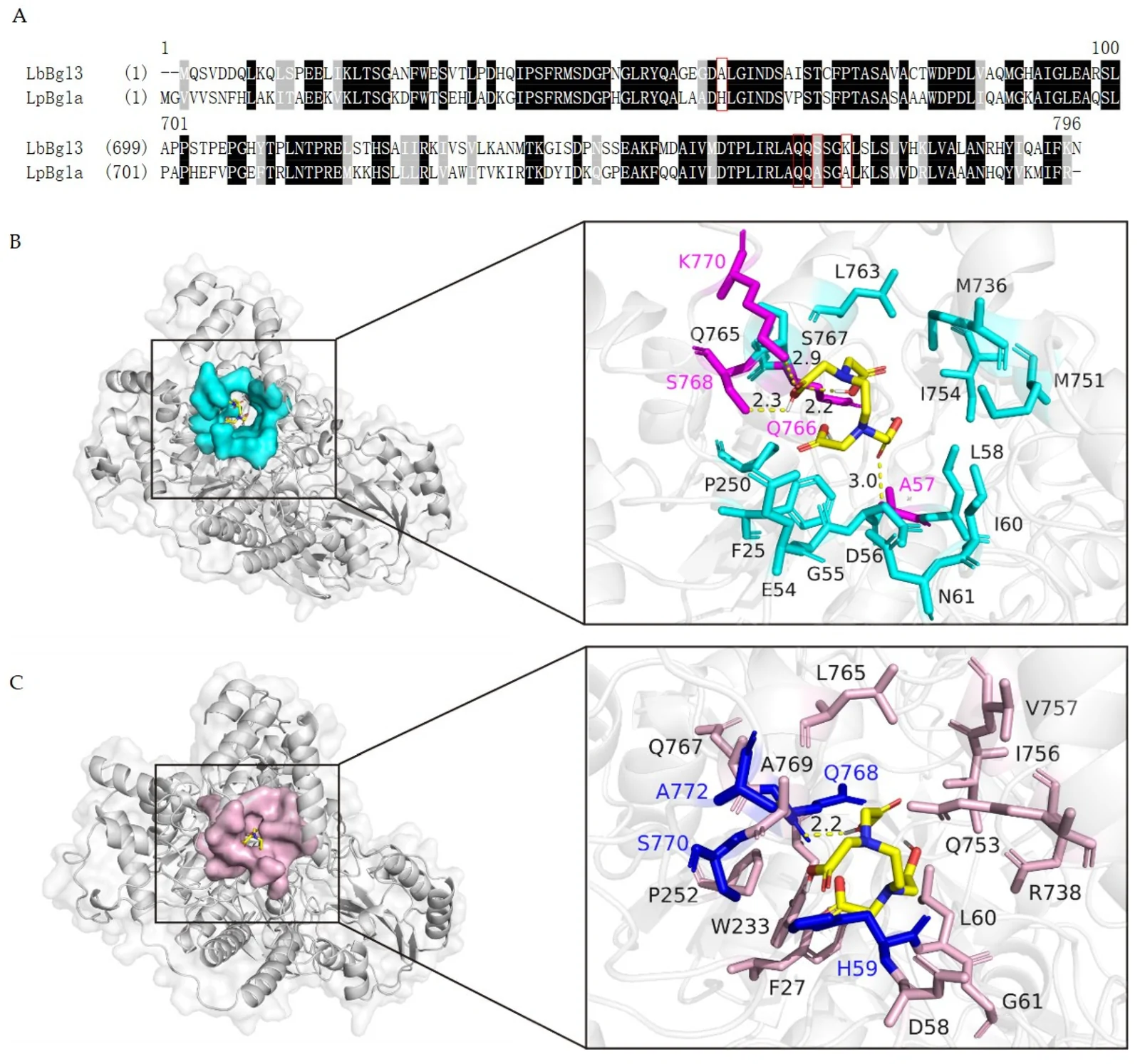
Sequence and structural differences in enhancing enzyme thermal stability through EDTA. (**A**) Sequence alignment of *LbBgl3* and LpBgla. Differential sites marked with red boxes. (**B**) Molecular docking of EDTA with *LbBgl3*, and the cyan sticks represent amino acid residues located within 6 Å distance of EDTA. The magenta sticks represent amino acid binding residues of *LbBgl3* (**C**) Molecular docking of EDTA with LpBgla. The light magenta sticks represent amino acid residues located within 6 Å distance of EDTA, the blue sticks represent aligned residues of LpBgla, the yellow sticks represent EDTA, and the yellow dotted line represents hydrogen bonds.

To elucidate the mechanism underlying the EDTA-induced thermostability enhancement of *LbBgl3*, molecular docking simulations were performed to predict potential intermolecular interactions between EDTA and *LbBgl3*. The docking results indicated a favorable binding affinity between EDTA and *LbBgl3*, with a calculated binding energy of −4.3 kcal mol^−1^ (Figure 7). Specifically, EDTA forms hydrogen bonds with four amino acid residues of *LbBgl3*, namely Ala57, Gln766, Ser768 and Lys770, which are located near the substrate-binding pocket. Hydrogen bonding networks in protein secondary structures are critical determinants of thermal stability, as these bonds serve to tether conformationally labile surface regions to the structurally stable core domain, thereby significantly strengthening the overall protein structure [35]. Studies have shown that thermophilic proteins contain more hydrogen bonds than their mesophilic homologs [36]. A plausible molecular-level explanation is that the hydrogen bonding interactions between EDTA and its binding residues effectively stabilize the conformational dynamics of the region surrounding the substrate-binding pocket, thereby alleviating the intrinsic thermolability of *LbBgl3*. Furthermore, LpBgla, a *β*-glucosidase from *L. paracasei* TK1501, shares 57.8% sequence identity with *LbBgl3*, but its enzymatic activity is inhibited in the presence of EDTA. Three-dimensional structural alignment of these two enzymes, combined with molecular docking analyses between each enzyme and EDTA, revealed that the His59, Ser770 and Ala772 residues of LpBgla do not form hydrogen bonds with EDTA. Collectively, these findings further support that the hydrogen bond network formed between EDTA and amino acid residues in the substrate-binding pocket is essential for stabilizing the overall conformation of *LbBgl3* and mitigating its inherent thermal instability.

### 3.5. Isothermal Titration Calorimetry Reveals Specific Binding Between EDTA and LbBgl3

Given the absence of metal-binding motifs or cofactor requirements in the *LbBgl3* sequence, and the fact that EDTA’s enhancing effect did not correlate with the removal of any specific metal ion (as the enzyme remained fully active in metal-free buffer), we opted for ITC to directly probe the EDTA-*LbBgl3* interaction rather than pursuing metal content analysis. This approach directly validates the molecular interaction underlying the observed stabilization, as metal content alone would not clarify whether EDTA exerts its effect through metal chelation or through direct binding to the protein.

**Figure 8:**
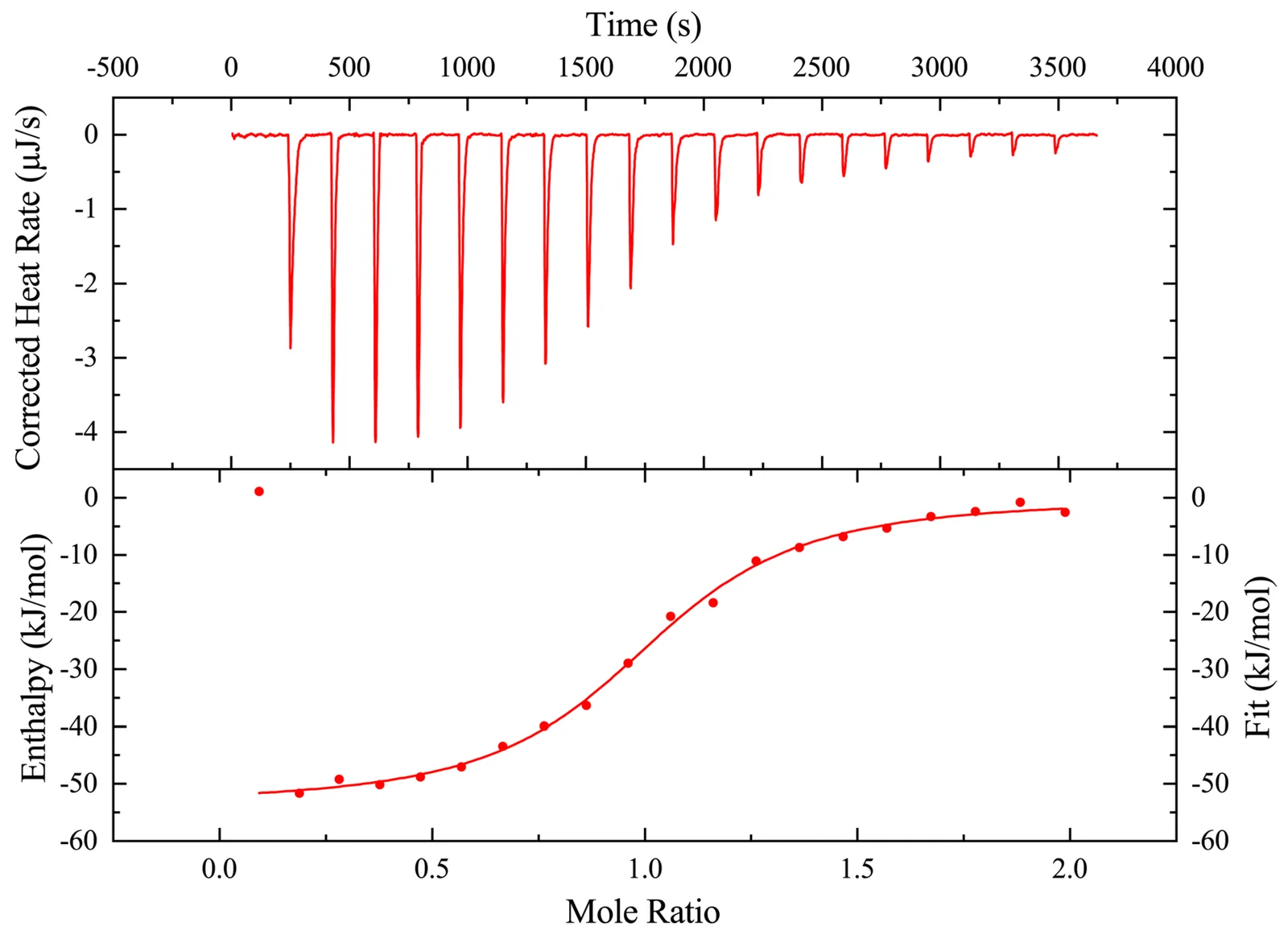
Isothermal titration calorimetry profile for the titration of EDTA against *LbBgl3*. Titrations were carried out at 298 K. Data are presented as mean ± SD from three independent biological replicates (n = 3).

ITC was utilized to directly verify the binding interaction between EDTA and *LbBgl3*, and the corresponding results are presented in Figure 8. The titration curves revealed that *LbBgl3* specifically binds EDTA at a molar ratio of 1:1, with *K_d_* of 1.33 μM, indicative of a relatively strong binding affinity. The binding process was strongly exothermic with Δ*H* of −53.75 kJ·mol^−1^, accompanied by reduced Δ*S* = −67.79 J·mol^−1^·K^−1^, reflecting increased ordering of the system upon complex formation. The overall Δ*G* was calculated to be −33.55 kJ·mol^−1^, demonstrating that the binding reaction occurs spontaneously. Hydrogen bonds, electrostatic interactions, and van der Waals forces dominate the intermolecular driving forces underlying this binding event. Complementary ITC experiments verified the spontaneous 1:1 specific binding between EDTA and *LbBgl3* with strong affinity, which follows an enthalpy-driven mechanism governed by hydrogen bonds, electrostatic and van der Waals interactions; this stable complex formation rationalizes the EDTA-mediated improvement in *LbBgl3* thermostability predicted by molecular docking.

While metal content analysis is commonly used to evaluate EDTA-mediated metal chelation, the absence of metal dependency and the unique enhancement pattern observed for *LbBgl3* suggested a non-classical mechanism. ITC was therefore employed to directly confirm the EDTA-*LbBgl3* binding interaction, providing unequivocal evidence for the direct interaction that underpins the observed thermostability enhancement.

## 4. Conclusions

This study presents the first systematic characterization of a GH3 family *β*-glucosidase, *LbBgl3*, from *L. buchneri*, elucidating its enzymatic characteristics, catalytic active sites, and key structural features. Biochemical characterization identifies *LbBgl3* as a cold-adapted, moderately acidophilic *β*-glucosidase with tolerance to low concentrations of salt and methanol, as well as high specific activity, highlighting *L. buchneri* as a promising source of novel biocatalysts. A notable finding is the enhancement of *LbBgl3* activity and thermostability by EDTA, mediated through hydrogen-bonding interactions that stabilize flexible conformations near the substrate-binding pocket. Future work should employ protein engineering, rational design, and artificial intelligence-aided design to improve its thermostability, thereby facilitating the development of novel, low-carbon, and green industrial applications based on this enzyme.

## Figures and Tables

**Table 1:** Enzymatic properties and applications of GH3 family *β*-glucosidase from *Lactobacillus*.

Strains	Enzyme Names	MW (kDa)	Optimal pH	Optimal Temperature (℃)	Specific Activity (U mg^−1^)	Main Characteristics	References
*Lentilactobacillus buchenri*	*LbBgl3*	86	5	37	799.67	Cold-adapted enzyme: Tolerant to low-salt/low-methanol solutions, poor thermal stability, and EDTA-enhanced thermal stability.	This study
*Lactobacillus antri*	rBGLa	81	6	45	142.00	Gardenoside → genipin	[25]
*L. brevis*	Bgy1	126	6	30	NR	Gypenoside XVII → Gypenoside LXXVI → CK	[7]
*L. casei TISTR* 1463	NR	75	4.5	35	3.05	EDTA inhibits *β*-glucosidase. *β*-glucosidase produces substances rich in antioxidants from anthocyanin-containing alternative crops.	[26]
*L. casei* SC1	NR	78	7.5	30	10.67	Hydrolysis of isoflavone glycosides	[15]
*L. ginsenosidimutans* EMML 3041T	BglL.gin-952	131	7.5	55	NR	EDTA exerts weak inhibition on *β*-glucosidase. Rb1 → Rd → Rg3(S)	[27]
*L. kimchi* JB301	NR	54	5	30–40	1.17	Dihydrostilbene glucoside hydrolysis activity (isorhynchophyllin glucoside, polydatin, moracin A, oxyresveratrol-3-O-glucoside). 100% conversion of polydatin to resveratrol	[28]
*L. paracasei* TK1501	LpBgla	87	5.5	30	3.00	In the presence of EDTA, the activity of LpBgla was significantly decreased. Hydrolysis of geniposide, salicin, polydatin, aesculin and arbutin into low-molecular-weight compounds.	[13,29]

NR: Not reported in the references.

**Table 2:** Amino acid compositions of *LbBgl3* and other *β*-Glucosidases with divergent temperature adaptation profiles.

Amino Acid	Proportion (%)			
	*LbBgl3*	M-GH3_A	MtBgl85	Bgl3B
Ala (A)	10.71	10.00	11.40	7.73
Arg (R)	3.53	2.88	5.31	3.57
Asn (N)	5.67	5.00	3.50	6.90
Asp (D)	4.91	5.88	5.18	6.06
Cys (C)	0.63	0.88	0.13	1.07
Gln (Q)	5.42	4.50	5.05	3.45
Glu (E)	5.67	6.88	6.61	4.64
Gly (G)	7.43	7.50	8.68	11.18
His (H)	1.76	2.13	2.20	1.31
Ile (I)	5.16	5.00	5.31	4.16
Leu (L)	9.82	9.88	10.49	6.90
Lys (K)	4.91	5.88	4.15	3.69
Met (M)	2.02	2.25	2.72	1.90
Phe (F)	2.90	4.00	3.76	3.33
Pro (P)	6.05	4.63	5.44	5.71
Ser (S)	7.18	5.75	5.70	7.02
Thr (T)	5.42	6.38	5.31	6.54
Trp (W)	1.13	1.13	1.42	2.85
Tyr (Y)	3.15	3.00	2.98	4.28
Val (V)	6.55	6.50	4.66	7.73

**Table 3:** The influence of metal ions and chemical reagents on *LbBgl3*.

Reagents (5 mM)	Relative Activity (%)	Reagents (5 mM/5%)	Relative Activity (%)
CK	100.00 ± 0.35		
Na^+^	105.29 ± 1.01	Mn^2+^	61.83 ± 1.45
K^+^	56.92 ± 6.75	Pb^2+^	34.86 ± 0.90
Li^+^	144.28 ± 4.09	Fe^2+^	0.57 ± 0.31
Ca^2+^	130.00 ± 8.10	Fe^3+^	14.70 ± 0.28
Mg^2+^	93.03 ± 3.31	EDTA	122.54 ± 1.48
Cd^2+^	110.77 ± 1.58	SDS	63.89 ± 1.71
Cu^2+^	72.97 ± 1.27	Methyl alcohol	110.48 ± 5.49
Co^2+^	131.56 ± 0.82	Ethyl alcohol	107.02 ± 0.86
Zn^2+^	109.04 ± 2.41	Isopropanol	51.57 ± 0.56
Ni^2+^	126.39 ± 2.61	*β*-mercaptoethanol	68.65 ± 1.28

## Data Availability

The original contributions presented in this study are included in the article. Further inquiries can be directed to the corresponding authors.
